# Lapatinib potentiates cytotoxicity of  YM155 in neuroblastoma via inhibition of the ABCB1 efflux transporter

**DOI:** 10.1038/s41598-017-03129-6

**Published:** 2017-06-08

**Authors:** Branka Radic-Sarikas, Melinda Halasz, Kilian V. M. Huber, Georg E. Winter, Kalliopi P. Tsafou, Theodore Papamarkou, Søren Brunak, Walter Kolch, Giulio Superti-Furga

**Affiliations:** 1 CeMM Research Center for Molecular Medicine of the Austrian Academy of Sciences, Vienna, Austria; 2 Systems Biology Ireland, University College Dublin, Belfield, Dublin, Ireland; 3 School of Medicine, University College Dublin, Belfield, Dublin, Ireland; 4 Conway Institute of Biomolecular & Biomedical Research, University College Dublin, Belfield, Dublin, Ireland; 5 Center for Biological Sequence Analysis, Department of Systems Biology, Technical University of Denmark, Lyngby, Denmark; 6 School of Mathematics and Statistics, University of Glasgow, Glasgow, United Kingdom; 7 Center for Physiology and Pharmacology, Medical University of Vienna, Vienna, Austria

## Abstract

Adverse side effects of cancer agents are of great concern in the context of childhood tumors where they can reduce the quality of life in young patients and cause life-long adverse effects. Synergistic drug combinations can lessen potential toxic side effects through lower dosing and simultaneously help to overcome drug resistance. Neuroblastoma is the most common cancer in infancy and extremely heterogeneous in clinical presentation and features. Applying a systematic pairwise drug combination screen we observed a highly potent synergy in neuroblastoma cells between the EGFR kinase inhibitor lapatinib and the anticancer compound YM155 that is preserved across several neuroblastoma variants. Mechanistically, the synergy was based on a lapatinib induced inhibition of the multidrug-resistance efflux transporter ABCB1, which is frequently expressed in resistant neuroblastoma cells, which allowed prolonged and elevated cytotoxicity of YM155. In addition, the drug combination (i.e. lapatinib plus YM155) decreased neuroblastoma tumor size in an *in vivo* model.

## Introduction

As a consequence of aggressive therapies, many childhood cancer survivors experience long-term adverse effects that negatively influence their quality of life. Neuroblastoma (NB) is the most common cancer in infancy^1^ and the most common extracranial solid tumor in children^2–4^ which can occur anywhere along the sympathetic nervous system. It is a remarkably heterogeneous disease, in both clinical behavior and genotype^5^. The main prognostic factors are the status of the *MYCN* (v-Myc avian myelocytomatosis viral oncogene neuroblastoma derived homolog) and *NTRK1* expression (encoding TRKA, a member of the TRK family of neurotrophin receptors), predisposing to poor and good prognosis, respectively. Furthermore, a range of different chromosomal aberrations are considered for risk stratification. It has been suggested that a major cause of therapeutic failure in neuroblastoma is the occurrence of resistance^6^. Overexpression of drug efflux transporters is one of the most common mechanisms of resistance^7^. In support of this hypothesis a number of ATP-binding cassette transporters (ABC transporters), including the multidrug-resistance transporter ABCB1, are known to be transcriptionally regulated by MYCN in neuroblastoma cells^8, 9^, thus supporting a drug-resistance role of ABCB1 in this tumor type. Overexpression of ABC transporters leads to intrinsic resistance to various therapeutics^10, 11^ and it is associated with poor clinical outcome^12^. Combination therapies can overcome these issues. A synergistic drug combination is more potent than equally effective doses of its components^13^, thus providing additional benefit to a patient over a simple increase in single component dosages. In this study, using a combinatorial screening approach, we identified a combination of the cytotoxic anticancer compound YM155 and the kinase inhibitor lapatinib to act highly synergistically in neuroblastoma including cells resistant to YM155. Further mechanistic studies revealed the ABCB1 transporter as a molecular determinant for this newly discovered synergy which we found to be conserved across neuroblastoma subtypes. We show that both intrinsic and/or acquired resistance to YM155 could be reverted by the efflux inhibition by lapatinib.

## Results

### Combinatorial screen reveals high degree of synergy between YM155 and lapatinib in neuroblastoma regardless of the MYCN and TRKA status

**Figure 1:**
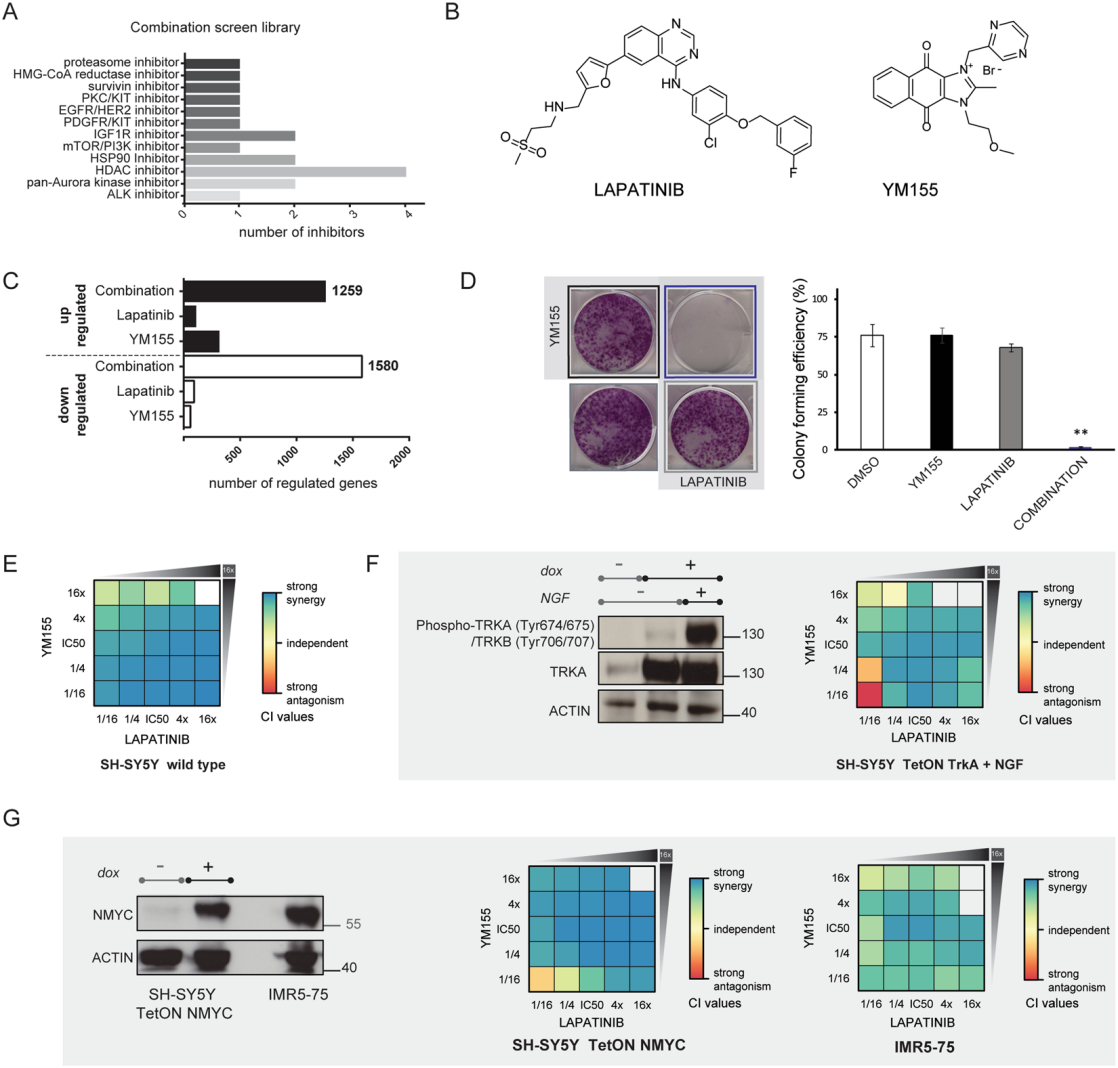
YM155 and lapatinib show high degree of synergy in neuroblastoma regardless of the MYCN and TRKA status. (**A**) Overview of the drug library used in the concentration matrix-based combination screen. (**B**) Chemical structures of lapatinib and YM155. (**C**) Microarray-based gene expression profiling. Bar graphs are showing the number of significantly upregulated and downregulated genes in each condition. (**D**) Colony formation assay; YM155 (black border) was used at 1/16 IC50 concentration (15 nM), lapatinib (gray border) at 1/8 IC50 (1 µM). Combination treatment (blue border): 15 nM YM155 and 1 µM lapatinib. DMSO (no border) treated cells were used as a control. Data shown as mean ± s.e.m. and images are representative of three individual experiments (ANOVA, **P < 0.01). (**E**) Heatmap of CI values in the YM155 and lapatinib combination matrix, indicating a strong synergistic interaction. Data represent the mean of triplicates. (**F**,**G**) Synergistic effect is preserved in *NTRK1* and *MYCN* highly expressed state. (**F**) Left, overexpression and activation of TRKA signaling in SH-SY5Y; right, heatmap of CI values in the combination matrix; data represent the mean of triplicates. (**G**) Left, overexpression of *MYCN* in SH-SY5Y and levels of MYCN protein in the IMR5–75 *MYCN* amplified cell line; right, heatmap of CI values in the combination matrix; data represent the mean of triplicates.

Following a previously established concentration matrix-based combinatorial drug screen approach (Winter *et al*.^14^) we tested a carefully selected library comprised of targeted anticancer agents proved to be safe in patients or already approved for therapeutic use in pairwise combinations using SH-SY5Y neuroblastoma cells line as a model system (Fig. 1A, Table S1). First, we assessed the IC50 values for the initial drug library in SH-SY5Y. Next, we selected a small combinatorial sub-library of drugs that potently inhibited cancer cell growth, because of the rapid augmentation of the number of data points with every compound in a concentration matrix–based screening design where each drug is combined with another (a total of 18 compounds; Fig. 1A, Table S1). The type of drug-drug interaction was assessed using the combination index (CI) method^15^ a quantification of the Loewe additivity approach^16^. Analyzing 153 drug combinations in five concentration points each, we found a strong synergy between the dual EGFR/HER2 kinase inhibitor lapatinib and the “survivin suppressant” sepantronium bromide (YM155) (Fig. 1B). In 2007, lapatinib (Tykerb/Tyverb^®^) was approved by the FDA for combination therapy in breast cancer and in 2010 for HER2 positive metastatic breast cancer. It was also in phase II clinical trials for malignant conditions in young patients such as refractory CNS malignancies^17^. YM155 causes downregulation of survivin and the induction of DNA damage^18, 19^, and it was evaluated in various clinical trials for single and combination therapy in prostate cancer, lymphoma and melanoma^20, 21^.

Using a microarray-based gene expression profiling approach, we observed marked global expression changes upon combinatorial treatment in SH-SY5Y, but little or no significantly regulated genes when SH-SY5Y cells were treated with either drug alone (Fig. 1C). We confirmed the synergy seen on a transcriptional level in a long-term colony formation assay in the SH-SY5Y cell line (Fig. 1D), where the compounds were applied at 10- or 15-fold lower concentrations than their respective IC50s (IC50 for YM155 in SH-SY5Y was 249 nM, while IC50 for lapatinib was 6.8 µM). Not surprisingly, single drug treatments at these concentrations were unable to cause cell death. However, the combination significantly inhibited colony formation and had a profound effect on cell viability. Moreover, the synergy occurred in the lower, clinically relevant dose range, at a concentration of lapatinib that is achievable in pediatric patients and considerably lower than the serum limit^22–24^. Whereas lapatinib is an orally active drug, the pharmacokinetic profile of YM155 is less favorable; still, the plasma concentration that corresponds to the concentration of YM155 where we observed high synergy is achievable and well tolerated in adult patients^20^. In contrast to *MYCN* overexpressing tumors, neuroblastomas that express *NTRK1* are likely to regress spontaneously or differentiate, if the TRKA ligand nerve growth factor (NGF) is expressed in the tumor^25^. Thus, a promising approach to induce spontaneous regression is via TRKA pathway stimulation^26^. When we treated the SH-SY5Y cells expressing *NTRK1* ^27^ with the two drugs, and stimulated cells with NGF, the synergy between YM155 and lapatinib was again detected, although slightly reduced compared to the wild type cells (Fig. 1E and F). These results suggest that the differentiation and apoptosis promoting functions of NGF overlap with the effects of the drug combination YM155 and lapatinib. The *MYCN* proto-oncogene is frequently amplified in neuroblastoma and correlates with advanced disease stage as well as aggressiveness and a poor prognosis^22, 24, 26^. The synergy screen was performed using the SH-SY5Y wild type cells which have no *MYCN* gene amplification. Thus, to test whether activation of *MYCN* affects the synergy observed for lapatinib and YM155 we used a genetically modified SH-SY5Y derivative cell line that allows for the inducible overexpression of this gene. The high degree of synergy was retained in *MYCN* overexpressing SH-SY5Y cells, and importantly, we detected a synergistic effect in the patient-derived and *MYCN*-amplified IMR5–75 cell line (Fig. 1G). The synergy between lapatinib and YM155 occurred regardless of the *MYCN* and *NTRK1* status.

### Synergistic effect is a consequence of a blocked efflux of YM155 by lapatinib

**Figure 2:**
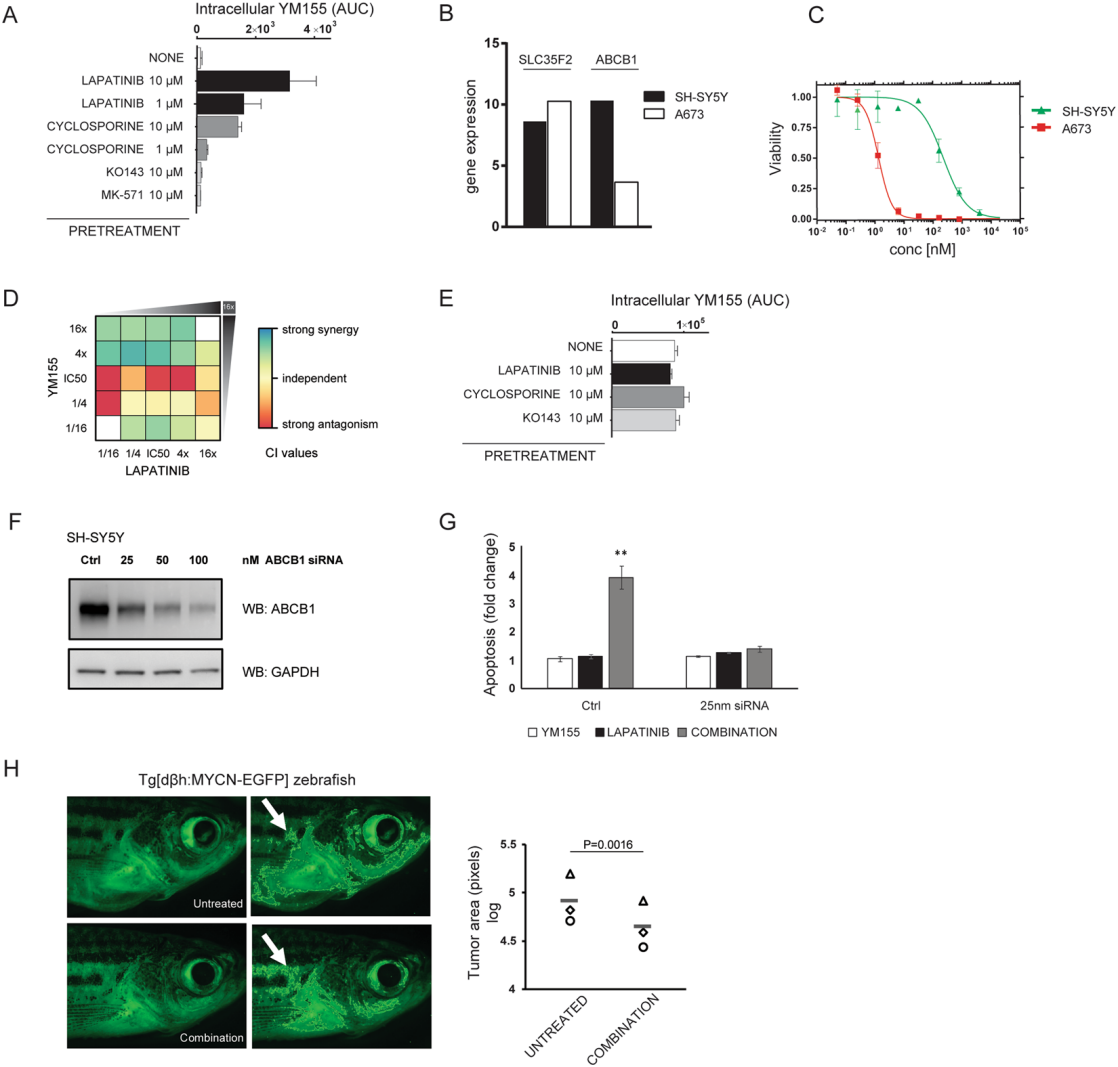
Synergistic effect is a consequence of a blocked efflux of YM155 by lapatinib *in vitro*. The anti-tumor effect of the combination is confirmed in an *in vivo* model. (**A**) Multiple-reaction monitoring assay in the SH-SY5Y neuroblastoma cells. Lapatinib inhibits the ABCB1 transporter and allows higher intracellular concentration of YM155. Area under the curve (AUC) corresponds to intracellular YM155 levels. Cyclosporine, MK-571 and KO143 are inhibitors of ABCB1, ABCC1 and ABCG2 transporters, (respectively). Data are the mean ± s.d. of triplicates. (**B**) Comparison of mRNA expression profiles of SLC35F2 and ABCB1 available in CCLE^32^ between SH-SY5Y and A673 cells. (**C**) Dose response curves of the effects of YM155 on the viability of SH-SY5Y (green) and A673 cells (red). (**D**) Heatmap of CI values in the combination matrix of YM155 and lapatinib in A673 cells. Data represent the mean of triplicates. (**E**) Multiple-reaction monitoring assay in A673 cells. Area under the curve (AUC) corresponds to intracellular YM155 levels. Cyclosporine and KO143 are inhibitors of ABCB1 and ABCG2 transporters, (respectively). Data are the mean ± s.d. of triplicates. (**F**) Efficiency of ABCB1 knock down after 24 hours. (**G**) Combined effect of ABCB1 knockdown and YM155 and/or lapatinib treatment on apoptosis of SH-SY5Y cells. Apoptosis was measured in SH-SY5Y cells which were transfected with control siRNA or siRNA against ABCB1 (25 nM); and treated with DMSO, 1 μM lapatinib and/or 15 nM YM155 for 48 hours. Percentages of cell death were used to calculate the fold changes in apoptosis of siRNA transfected and drug treated cells compared to untreated siRNA transfected cells. (Mean ± SEM, n = 3, **P < 0.01, ANOVA). (**H**) Analysis of neuroblastoma growth in *Tg[dβh:MYCN-EGFP]* zebrafish, expressing MYCN-EGFP under the control of dopamine-β-hydroxylase promoter. Fish were treated with lapatinib and YM155 (2 µM and 6.5 nM, respectively) for 7 days. Zebrafish were imaged before and after treatment with the same settings. The fluorescent area behind gills corresponding to tumors (white arrow) were measured by using ImageJ (representative images of three independent experiments, n = 3). A quantitation of the results is shown in the graph, where each symbol represents a fish (P = 0.0016, paired t test).

In order to examine whether EGFR/HER2 inhibition is required for the synergistic effect with YM155, we used other EGFR or EGFR/HER2 inhibitors instead of lapatinib. Interestingly, only some of them acted similarly, while others did not result in a synergistic effect (data not shown). This was suggestive of the fact that the inhibition of the EGFR/HER2 signaling pathway was not critical for the response. Therefore, we investigated alternative possibilities. Lapatinib was shown to inhibit both ABCB1 and ABCG2 transporters^28^, and YM155 is a substrate of ABCB1^29^. Moreover, neuroblastoma cell lines resistant to YM155 were shown to express high levels of ABCB1^30^. Neuroblastomas are known to express high levels of ABCC1 transporter as well^6, 31^. Thus we tested whether lapatinib affects the intracellular concentration of YM155, using a mass spectrometry based multiple reaction-monitoring assay (MS-MRM) (Fig. 2A). We found that treatment with lapatinib increased the intracellular concentration of YM155 even to a larger extent than cyclosporine, which is a known inhibitor of ABCB1. Conversely, pre-treatment with inhibitors of the ABCC1 and ABCG2 transporters (MK-571 and KO143, respectively), did not influence the intracellular YM155 levels. These data suggest that the synergy may stem from lapatinib inhibiting ABCB1 and thereby enabling the intracellular accumulation of YM155. We, therefore, tested the lapatinib/YM155 combination in A673 Ewing sarcoma cells, as Ewing sarcoma cells in general have low expression of ABCB1 as reported in the Cancer Cell Line Encyclopedia data (CCLE)^32^. Both SH-SY5Y and A673 express similar amounts of SLC35F2, a solute carrier transporter that is responsible for YM155 influx^18^ (Fig. 2B). Thus, the cell lines are expected to have similar capacity to uptake YM155, but a different ability to eliminate it via the ABCB1 transporter. Indeed, when we compared the IC50s of YM155 in SH-SY5Y and A673 cells we noted a dramatic difference in sensitivity, with values of ~1 nM in A673 but ~250 nM in SH-SY5Y, which can be attributed to differences in levels of ABCB1 between the two cell lines (Fig. 2C). Furthermore, we observed a mild to strong antagonism between the two compounds in Ewing sarcoma cell line (Fig. 2D). Directly assessing the intracellular concentration of YM155 using the MS-MRM assay we found that neither lapatinib, KO143, nor cyclosporine could influence the intracellular concentration of YM155 in A673 (Fig. 2D). Thus, the strong synergistic effect observed when lapatinib and YM155 were combined was inherent to ABCB1 expressing neuroblastoma cells, where ABCB1 inhibition by lapatinib augmented YM155 concentrations and cytotoxicity. Finally, we performed a gene functional study, where ABCB1 expression in SH-SY5Y cells was downregulated by siRNA in order to assess whether the drug synergy was dependent on ABCB1 (Fig. 2F). Unlike in cells that express high levels of ABCB1, ABCB1 knockdown dramatically suppressed the synergistic effect since similar percentages of apoptotic cells were observed in the single drug treated groups and after the combinatorial treatment (Fig. 2G). Thus, we concluded that the enhanced cytotoxicity of YM155 is due to the lapatinib interference with the ABCB1 transporter.

### The anti-tumor effect of the combination was confirmed *in vivo*

To investigate whether the drug combination is effective *in vivo*, we tested the combination in a transgenic zebrafish model, where the overexpression of human *MYCN* in the peripheral sympathetic nervous system induces tumors in the fish analog of the adrenal medulla^33^. These tumors closely resemble human neuroblastoma, and the model has been successfully used to study interactions between *MYCN* and other oncoproteins and the role of developmental pathways in neuroblastoma pathogenesis^34, 35^. We exposed neuroblastoma bearing fish to YM155 and lapatinib for seven days and observed that the tumor size was considerably smaller after the treatment (Fig. 2H). These results prompt a further clinical evaluation of this novel combination.

## Discussion

YM155 is an anticancer drug which causes DNA damage and survivin downregulation, and therefore was considered a promising treatment option in neuroblastoma^30, 36, 37^. However, neuroblastoma cells with high ABCB1 levels were intrinsically resistant to YM155^30^. ABC transporters are often upregulated in the most aggressive neuroblastoma variants in response to treatment, causing acquired resistance and ultimately leading to therapy ineffectiveness^12, 31^. Performing a factorial matrix-design combinatorial drug screen on the SH-SY5Y neuroblastoma cells line we found that the FDA approved EGFR/HER2 tyrosine kinase inhibitor lapatinib strongly synergized with YM155. We showed that the synergy occurs in TRKA overexpressing as well as in MYCN overexpressing and amplified neuroblastoma cells. The mechanistic basis for the synergy is the blockade of the ABCB1 efflux pump by lapatinib. Consequently, the intracellular concentration of YM155 was dramatically increased and its effect potentiated and prolonged upon combination with lapatinib. Moreover, this strong synergy was absent in a low-ABCB1 expressing Ewing sarcoma cells line. Importantly, the strongest response was observed at concentrations much lower than the corresponding *in vitro* IC50 values each individual drug. Such concentrations are achievable and well tolerated in patients for both agents^20, 23^. Using a zebrafish model system that reproduces human *MYCN* amplified, aggressive neuroblastoma, we could confirm the anti-tumor effect of the combination in an *in vivo* model. Therefore, the combination of lapatinib and YM155 is a promising treatment option for neuroblastoma with either intrinsic or acquired ABCB1 upregulation. The elucidation of the mechanistic basis for the synergy, i.e. lapatinib inhibiting ABCB1 allowing YM155 to accumulate, also provides a potential biomarker for stratifying patients for treatment. Furthermore, our results suggest that lapatinib should be considered as combination therapy for other agents that are eliminated from cells by the ABCB1 transporters.

## Materials and Methods

### Viability assays

The individual drug effects were determined in proliferation assays using Cell Titer Glo (Promega Inc., Madison WI, USA) basically as described previously^14^. 4 × 10³ cells were seeded in 96-well plates in triplicates and treated with drugs 24 h after plating. Serial dilutions in a range between 20 µM and 0.05 µM were applied for 72 hours. IC50 values were determined by fitting a dose response curve to the data points using non-linear regression analysis utilizing the GraphPad Prism software.

### Compounds

Lapatinib was purchased from LC Laboratories (Woburn, MA, USA). YM155 was obtained from Selleck Chemicals (Selleckchem, Houston, TX, USA). The full compound library with their respective manufacturers is given in Supplementary Table S1.

### Determination of synergy and analysis of the screen

The combination index (CI) was used for the quantification of synergistic, antagonistic or additive effects of each drug pair as described previously^38^. In order to capture any synergistic effect, we generated duplicate matrices for each drug pair, positioned in different screening plates. Cell viability was measured across a range of dose levels for each drug pair without maintaining the ratio of dose levels constant. In its conventional terminology, a non-constant ratio experimental design has been deployed. Such an experimental setup has been selected for the purposes of attaining a broad screening window across drug combinations and concentrations levels. A well-known trade-off of a non-constant dose ratio experimental design is the lack of ability to simulate CI across varying dose levels to estimate a smooth dose-response curve. However, it is possible to calculate CI for each combination data point under the non-constant ratio design. The evaluated CI has then been mainly employed for classifying drug pairs as synergistic or antagonistic with respect to CI cut-offs, thus allowing to detect drug combinations with non-additive effects. Estimating CI for a given drug pair at an observed point entails a two-step process. Initially, the kinetic order m and median effect dose *D*_*m*_ appearing in median-effect equation (1) are estimated for each single drug. The median-effect equation expresses as

1 $$\frac{{f}_{a}}{{f}_{u}}={(\frac{D}{{D}_{m}})}^{m},$$

where the observables are the fraction of unaffected (viable) cells *f*_*u*_ and drug dose D, while *f*_*a*_ = 1 − *f*_*u*_ is the fraction of affected (inhibited) cells. To apply equation (1) to a single drug, the *f*_*a*_, *f*_*u*_, and D values for that drug are taken into account across all drug pairs at the instances of zero dosage for the other drug in each pair. Equation (1) is then log-transformed, and simple linear regression is used for estimating m and *D*_*m*_. The second step involves computing the combination index

2 $$CI=\frac{{D}_{1}}{{({D}_{m})}_{1}{(\frac{{f}_{a}}{{f}_{u}})}^{1-{m}_{1}}}+\frac{{D}_{2}}{{({D}_{m})}_{2}{(\frac{{f}_{a}}{{f}_{u}})}^{1-{m}_{2}}},$$

where *D*_*i*_, (*D*_*m*_)_*i*_ are the dose and estimated median dose for drug i, $i\in \{1,2\}$, in the pair.

### Microarrays

SH-SY5Y cells were treated with DMSO, 15 nM YM155, 1 µM lapatinib or the combination (15 nM YM155 and 1 µM lapatinib) for 6 hours, in triplicates. RNA was extracted using the Qiagen RNAeasy kit according to the manufacturer’s instructions. Total RNA was then used for GeneChip analysis. Preparation of terminally labeled cDNA, hybridization to genome-wide GeneChip Human Gene 1.0 ST Arrays and scanning of the arrays were done according to the manufacturer’s instructions (Affymetrix). For differential expression analysis, the aroma.affymetrix and the Bioconductor Limma packages were used. Empirical Bayes approach was applied, while the Benjamini and Hochberg method was used for false discovery control; a false discovery rate of 0.05 was selected, providing an adjusted p.value (q.value).

### Colony formation assay

1 × 10^4^ cells per well were seeded in six-well plates (in triplicates). Next day the cells were treated with either DMSO (equal to the highest amount of compound dilution, maximum 0.2%) or compounds at combination concentrations. Cells were incubated at 37 °C, 5% CO2 for 7–10 days. Medium was aspirated, cells were washed with PBS (Gibco), stained with crystal violet solution (0.5% in 6% glutaraldehyde) and left to dry overnight. For quantification of results, ultraviolet absorbance of crystal violet was determined at 570 nm following solubilization by 70% ethanol. Data were analyzed using the ANOVA single factor analysis followed by ad hoc Tukey test.

### Cell culture

The SH-SY5Y and SH-SY5Y *NTRK1* tetracycline (Tet)–inducible cell lines, SH-SY5Y *MYCN* tetracycline (Tet)–inducible, IMR5–75 cell lines and A673 cell lines were received as generous gifts from Drs. Frank Westermann, Johannes Schulte and Javier Alonso. A673 cells were cultured in DMEM (Sigma) media containing 10% fetal bovine serum and 10 U mL^−1^ penicillin/streptomycin (Gibco). SH-SY5Y and IMR5–75 cells were cultured in RPMI 1640 (Sigma) media containing 10% fetal bovine serum and 10 UmL^−1^ penicillin/streptomycin (Gibco).

### Antibodies used for immunoblotting

Rabbit anti-actin (AAN01) (Cytoskeleton, AAN01), mouse anti-tubulin (DM1A) (Abcam, ab7291), mouse GAPDH (Santa Cruz, sc-365062), mouse N-Myc (Santa Cruz, sc-53993), rabbit phospho-TRKA (Tyr674/675)/TrkB (Tyr706/707) (C50F3) (Cell Signaling, 4621S), rabbit TRK (C14) (Santa Cruz, sc-11).

### Determination of intracellular drug levels via MS-MRM

2 × 10^5^ cells were treated with drugs at 37 °C as indicated. Subsequently, cells were washed three times with ice-cold PBS and extracted directly with 300 μl 80% ice-cold methanol. Extracts were cleared by centrifugation for 20 min at 4 °C at 16,000 g, and supernatants were used for subsequent MS quantifications by MS (Waters Xevo TQ system). MRM settings were automatically generated for every compound using the IntelliStart software (Waters), and quantification was conducted on the basis of the intensity of three daughter ions.

### RNA Interference

The ABCB1 Silencer siRNA (siRNA ID: 4029) and non-targeting control siRNA were obtained from Ambion. Transfections were performed using the JetPrime reagent (Polyplus Transfection) according to the manufacturer’s instructions. Cells were lysed after 24 hours for Western blot analysis to test the efficiency of knockdown.

### Apoptosis Assay

After the drug treatments, cells were fixed in ethanol and stained with propidium iodide, as previously described^39^. Apoptosis was measured by identifying the sub-G1 population using an Accuri C6 flow cytometer (BD).

### Zebrafish experiments

Zebrafish experiments were approved by the UCD Animal Research Ethics Committee and the Healthcare Products Regulatory Authority (AE18982/P038). All methods were performed in accordance with the relevant guidelines and regulations. The transgenic *Tg[dβh:MYCN-EGFP]* zebrafish line was a generous gift from Prof. A. Thomas Look (Dana-Farber Cancer Institute, Harvard Medical School)^33^. Adult *Tg[dβh:MYCN-EGFP* *]* zebrafish (*Danio rerio*) were maintained on a 14 h light/10 h dark cycle at 28 °C; and monitored for fluorescent tumor masses every two weeks. Approximately 20% of fish develop the equivalent of human neuroblastoma by about 8 months of age^33^. Drug treatments of zebrafish were performed as described earlier^40^. Zebrafish developing tumors (age: 21 months; n = 3) were treated with YM155 and lapatinib every 72 hours for a total of seven days. Fish water was changed every 72 hours, and drugs were dissolved in the fish water. Zebrafish were anesthetized by 0.016% Tricaine prior to microscopic analysis. An Olympus SZX10 fluorescent stereo microscope equipped with an Olympus DP71 camera was used to capture images. The fluorescent area posterior to the gills, which corresponds to the region of neuroblastoma growth^33^, was analysed using the ImageJ software (v1.44p).

## Electronic supplementary material

Supplementary File and Dataset 1
