# Supplementary material for: Lapatinib potentiates cytotoxicity of YM155 in neuroblastoma via inhibition of the ABCB1 efflux transporter
Source: Sci Rep. 2017 Jun 8;7:3091. doi: 10.1038/s41598-017-03129-6 (PMC5465103; doi:10.1038/s41598-017-03129-6)
Supplement: Supplementary file 1 — Supplementary File and Dataset 1 [file 41598_2017_3129_MOESM1_ESM.zip › 20160908_Suppl Fig 1 & Suppl Table 1.pdf]

Lapatinib potentiates cytotoxicity of YM155 in neuroblastoma via inhibition of the ABCB1 efflux transporter

Branka Radic-Sarikas[1], Melinda Halasz[2,3], Kilian V. M. Huber[1], Georg E. Winter[1], Kalliopi P. Tsafou[5], Theodore Papamarkou[6], Søren Brunak[5], Walter Kolch[2,3,4] and Giulio Superti-Furga[1,7,*]

[1] CeMM Research Center for Molecular Medicine of the Austrian Academy of Sciences, Vienna, Austria

[2] Systems Biology Ireland, University College Dublin, Belfield, Dublin 4, Ireland

[3] School of Medicine, University College Dublin, Belfield, Dublin 4, Ireland

[4] Conway Institute of Biomolecular & Biomedical Research, University College Dublin, Belfield, Dublin 4, Ireland

[5] Center for Biological Sequence Analysis, Department of Systems Biology, Technical University of Denmark, Lyngby, Denmark

[6] School of Mathematics and Statistics, University of Glasgow, Glasgow, United Kingdom

[7] Center for Physiology and Pharmacology, Medical University of Vienna, Vienna, Austria

Supplementary Table S1. Overview of compound library and sub-library used in the initial and combinatorial screen. Given are the compound names, manufacturers, clinical trial status and IC50s in SH-SY5Y cell line.

Supplementary Figure S1. Full-length blots of p-TRKA, TRKA and NMYC, corresponding to Figure 1.

Supplementary Figure S1

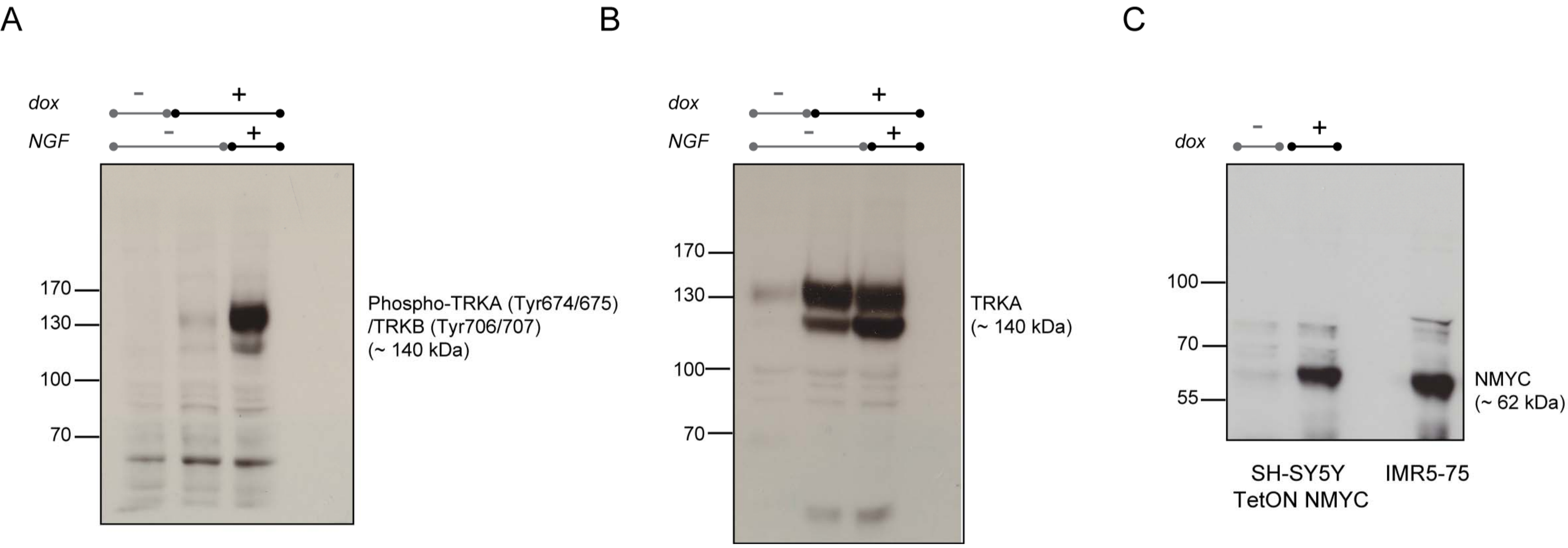

Supplementary Table S1.

Overview of compound library and sub-library used in the initial and combinatorial screen. Given are the compound names, manufacturers, clinical trial status and IC50s in SH-SY5Y cell line.

INITIAL SCREENING DRUG PANEL

	COMPOUND NAME	MAIN COGNATE TARGET	MANUFACTURER	CLINICAL STATUS (cancer related)	IC50 [nM] in SH-SY5Y
1	Bortezomib	proteasome inhibitor	Selleck Chemicals	approved May 2003	10
2	Imatinib	ABL Inhibitor	LC Laboratories	approved February 2002	5848
3	Nilotinib	ABL inhibitor	LC Laboratories	approved October 2007	10000
4	Dasatinib	ABL/SRC inhibitor	LC Laboratories	approved June 2006	10000
5	Bosutinib	ABL/SRC inhibitor	LC Laboratories	approved September 2012	5773
6	Bafetinib	ABL/LYN inhibitor	WuXi AppTec	phase II	6221
7	Gefitinib	EGFR Inhibitor	LC Laboratories	approved May 2003	6076
8	Erlotinib	EGFR inhibitor	Selleck Chemicals	approved November 2004	10000
9	Lapatinib	HER2/EGFR inhibitor	LC Laboratories	approved March 2007	6801
10	Sunitinib	PDGFR/KIT inhibitor	Selleck Chemicals	approved January 2006	5774
11	Sorafenib	PDGFR/BRAF inhibitor	Selleck Chemicals	approved December 2005	6332
12	PKC412	PKC/KIT inhibitor	LC Laboratories	breakthrough therapy designation February 2016	134
13	Pazopanib	VEGFR Inhibitor	LC Laboratories	approved April 2012	10000
14	OSI-906	IGF1R inhibitor	Selleck Chemicals	phase III	1135
15	BMS-754807	IGF1R inhibitor	Active Biochem	phase II	41
16	Crizotinib	ALK inhibitor	LC Laboratories	approved August 2011	434
17	NVP-BEZ235	PI3K/mTOR Inhibitor	Selleck Chemicals	phase II	245
18	Everolimus	FKBP12/mTOR inhibitor	Selleck Chemicals	approved April 2012	10000
19	Danusertib	pan-Aurora kinase inhibitor	LC Laboratories	phase II	1267
20	Tozasertib	pan-Aurora kinase inhibitor	LC Laboratories	phase II	63
21	Geldanamycin	HSP90 Inhibitor	AG Scientific	tool cmpd	39
22	17-AAG	HSP90 Inhibitor	InvivoGen	phase II, phase II/III combination study	1082
23	Atorvastatin	HMG-CoA reductase inhibitor	Selleck Chemicals	phase II	6619
24	MS-275	HDAC inhibitor	Selleck Chemicals	phase III	1664
25	SAHA	HDAC inhibitor	Selleck Chemicals	approved October 2006	1911
26	Belinostat	HDAC inhibitor	Selleck Chemicals	approved July 2014	265
27	Panobinostat	HDAC inhibitor	Selleck Chemicals	phase II	29
28	YM155	survivin inhibitor	Selleck Chemicals	phase II	249
29	Cyclopamine	Shh inhibitor	LC Laboratories	tool cmpd	10000
30	Vismodegib	Shh inhibitor	LC Laboratories	approved January 2012	10000
31	ATRA	RAR/RXR ligand	Selleck Chemicals	phase II	10000
32	DAPT	γ-secretase inhibitor	Selleck Chemicals	phase II	10000
33	Navitoclax	Bcl-2 inhibitor	Selleck Chemicals	phase II	6227

** the scale was truncated at 10000*

COMBINATORIAL SCREENING DRUG PANEL

	COMPOUND NAME	MAIN COGNATE TARGET	MANUFACTURER	CLINICAL STATUS (cancer related)	IC50 [nM] in SH-SY5Y
1	Bortezomib	proteasome inhibitor	Selleck Chemicals	approved May 2003	10.4
2	Lapatinib	HER2/EGFR inhibitor	LC Laboratories	approved March 2007	6801
3	Sunitinib	PDGFR/KIT inhibitor	Selleck Chemicals	approved January 2006	5774
4	PKC412	PKC/KIT inhibitor	LC Laboratories	breakthrough therapy designation February 2016	134.2
5	OSI-906	IGF1R inhibitor	Selleck Chemicals	phase III	1135
6	BMS-754807	IGF1R inhibitor	Active Biochem	phase II	40.5
7	Crizotinib	ALK inhibitor	LC Laboratories	approved August 2011	433.5
8	NVP-BEZ235	PI3K/mTOR Inhibitor	Selleck Chemicals	phase II	245.3
9	Danusertib	pan-Aurora kinase inhibitor	LC Laboratories	phase II	1267
10	Tozasertib	pan-Aurora kinase inhibitor	LC Laboratories	phase II	62.9
11	Geldanamycin	HSP90 Inhibitor	AG Scientific	tool cmpd	39
12	17-AAG	HSP90 Inhibitor	InvivoGen	phase II, phase II/III combination study	1082
13	Atorvastatin	HMG-CoA reductase inhibitor	Selleck Chemicals	phase II	6619
14	MS-275	HDAC inhibitor	Selleck Chemicals	phase III	1664
15	SAHA	HDAC inhibitor	Selleck Chemicals	approved October 2006	1911
16	Belinostat	HDAC inhibitor	Selleck Chemicals	approved July 2014	264.8
17	Panobinostat	HDAC inhibitor	Selleck Chemicals	phase II	29.26
18	YM155	survivin inhibitor	Selleck Chemicals	phase II	248.9

** the scale was truncated at 10000*

SUB-LIBRARY
